# A genome-wide association scan on estrogen receptor-negative breast cancer

**DOI:** 10.1186/bcr2772

**Published:** 2010-11-09

**Authors:** Jingmei Li, Keith Humphreys, Hatef Darabi, Gustaf Rosin, Ulf Hannelius, Tuomas Heikkinen, Kristiina Aittomäki, Carl Blomqvist, Paul DP Pharoah, Alison M Dunning, Shahana Ahmed, Maartje J Hooning, Antoinette Hollestelle, Rogier A Oldenburg, Lars Alfredsson, Aarno Palotie, Leena Peltonen-Palotie, Astrid Irwanto, Hui Qi Low, Garrett HK Teoh, Anbupalam Thalamuthu, Juha Kere, Mauro D'Amato, Douglas F Easton, Heli Nevanlinna, Jianjun Liu, Kamila Czene, Per Hall

**Affiliations:** 1 Department of Medical Epidemiology and Biostatistics, Karolinska Institutet, P.O. Box 281, Stockholm 17177, Sweden; 2 Human Genetics, Genome Institute of Singapore, 60 Biopolis St, Singapore 138672, Singapore; 3 Department of Biosciences and Nutrition, Karolinska Institutet, Hälsovägen 7-9, Novum, SE-141 81, Huddinge, Sweden; 4 Department of Obstetrics and Gynecology, Helsinki University Central Hospital, P.O. Box 700, 00029 HUS, Helsinki, Finland; 5 Department of Clinical Genetics, Helsinki University Central Hospital, Haartmanink 2 B, 00029 HUS, Helsinki, Finland; 6 Department of Oncology, Helsinki University Central Hospital, P.O. Box 180, 00029 HUS, Helsinki, Finland; 7 Department of Public Health and Primary Care, Strangeways Research Laboratory, University of Cambridge, Wort's Causeway, Cambridge CB1 8RN, UK; 8 Department of Oncology, Strangeways Research Laboratory, University of Cambridge, Wort's Causeway, Cambridge CB1 8RN, UK; 9 Department of Medical Oncology, Rotterdam Family Cancer Clinic, Erasmus University Medical Center, Daniel den Hoed Cancer Center, Groene Hilledijk 301, 3075 EA Rotterdam, Netherlands; 10 Department of Medical Oncology, Erasmus University Medical Center, Josephine Nefkens Institute, Dr. Molenwaterplein 50, 3015 GE Rotterdam, The Netherlands; 11 Department of Clinical Genetics, Rotterdam Family Cancer Clinic, Erasmus University Medical Center, Dr. Molenwaterplein 50, 3015 GE Rotterdam, Netherlands; 12 Institute of Environmental Medicine, Karolinska Institutet, P.O. Box 281, Stockholm 17177, Sweden; 13 Institute for Molecular Medicine Finland, FIMM, University of Helsinki, P.O. Box 20, FI-00014, Finland; 14 Public Health Genomics Unit, National Institute for Health and Welfare, P.O. Box 30, FI-00271 Helsinki, Finland; 15 Wellcome Trust Sanger Institute, Wellcome Trust Genome Campus, Hinxton, Cambridge, CB10 1SA, UK; 16 Program in Medical and Population Genetics, Broad Institute of Harvard and Massachusetts Institute of Technology, Cambridge, MA 02142, USA; 17 Clinical Research Centre, Karolinska Institute, Karolinska University Hospital Huddinge, SE-141 86, Huddinge, Sweden; 18 Department of Medical Genetics, University of Helsinki, Haartman Institute, P.O. Box 21 (Haartmaninkatu 3), FI-00014, Finland; 19 Folkhälsan Institute of Genetics, Folkhälsan Research Center; University of Helsinki, Haartmaninkatu 8, Biomedicum 1, P.O. Box 63, FI-00014, Finland

## Abstract

**Introduction:**

Breast cancer is a heterogeneous disease and may be characterized on the basis of whether estrogen receptors (ER) are expressed in the tumour cells. ER status of breast cancer is important clinically, and is used both as a prognostic indicator and treatment predictor. In this study, we focused on identifying genetic markers associated with ER-negative breast cancer risk.

**Methods:**

We conducted a genome-wide association analysis of 285,984 single nucleotide polymorphisms (SNPs) genotyped in 617 ER-negative breast cancer cases and 4,583 controls. We also conducted a genome-wide pathway analysis on the discovery dataset using permutation-based tests on pre-defined pathways. The extent of shared polygenic variation between ER-negative and ER-positive breast cancers was assessed by relating risk scores, derived using ER-positive breast cancer samples, to disease state in independent, ER-negative breast cancer cases.

**Results:**

Association with ER-negative breast cancer was not validated for any of the five most strongly associated SNPs followed up in independent studies (1,011 ER-negative breast cancer cases, 7,604 controls). However, an excess of small *P*-values for SNPs with known regulatory functions in cancer-related pathways was found (global *P *= 0.052). We found no evidence to suggest that ER-negative breast cancer shares a polygenic basis to disease with ER-positive breast cancer.

**Conclusions:**

ER-negative breast cancer is a distinct breast cancer subtype that merits independent analyses. Given the clinical importance of this phenotype and the likelihood that genetic effect sizes are small, greater sample sizes and further studies are required to understand the etiology of ER-negative breast cancers.

## Introduction

Breast cancer is a heterogeneous disease and can be characterized on the basis of estrogen receptor (ER) expression in the tumour cells. The two breast cancer subtypes (ER-positive and ER-negative) are generally considered as biologically distinct diseases and have been associated with remarkably different gene expression profiles [1,2]. ER status is important clinically, and is used both as a prognostic indicator and treatment predictor since it determines if a patient may benefit from anti-estrogen therapy. Approximately one-third of all breast cancers are ER-negative, and cancers of this ER subtype are highly age-dependent and generally have a more aggressive clinical course than hormone receptor-positive disease.

Estimates show that close to a third of the total risk of breast cancer may be attributed to heritable factors [3]. Several large-scale genome-wide single nucleotide polymorphism (SNP) association studies (GWAS) have identified multiple susceptibility loci for breast cancer [4-11], but it is estimated that the currently known common risk variants identified by this approach explains only 5.8% of the proportion of familial risk of breast cancer.

Aside from traditional agnostic SNP studies, pathway-based approaches have also emerged in the recent GWAS literature [12-20]. These novel methods have been developed to mine modest association signals from genome-wide SNP data using prior knowledge on biologically pathways and networks, and have the potential to complement traditional agnostic SNP approaches to provide fertile grounds for follow-up studies of both a genetic and molecular nature. Subtypes of breast cancer, to our knowledge, have not been studied using a pathway-based approach.

Although many of the SNPs identified for breast cancer through GWAS scans have been found to be more strongly associated with ER-positive disease than ER-negative disease [21,22], there is no quantitative assessment on whether breast cancers of the two different ER subtypes share a polygenic component. In this study, we performed a genome-wide association scan on 617 ER-negative cases and 4,583 controls, the first of its kind, and examined 285,984 SNPs for common variants and biological pathways associated with this unique subtype of breast cancer. We also searched for evidence that ER-negative breast cancer is distinct from ER-positive breast cancer by assessing the amount of shared polygenic variation between the two breast cancer subtypes.

## Materials and methods

Full methods accompany this paper in Additional file 1.

### Study populations used in the discovery stage

Table 1 summarizes the demographics of cases and controls used in this study. The discovery stage consists of cases and controls from Finland and Sweden. The validation stage consists of breast cancer cases from two further studies: the Study of Epidemiology and Risk factors in Cancer Heredity (SEARCH) and Rotterdam Breast Cancer Study (RBCS) (1,011 ER-negative cases, 7,604 controls), both previously described in Lesueur *et al. *[23]. Informed consent was obtained from all subjects. For all populations, blood samples were obtained from individuals according to protocols and informed-consent procedures approved by institutional review boards.

**Table 1:** Summary of samples and genotyping platforms used in the discovery and validation stages

Stage	Study	Type	No. of samples after quality control	Genotyping platform
Discovery	Swedish	ER-negative cases	153	HumanHap300 supplemented by HumanHap240S
		Controls	764	HumanHap550
		Additional controls from EIRA study	650	HumanHap300
				
	Finnish	ER-negative cases	226	HumanHap550
		ER-negative cases	238	Quad610 (v1)
		Controls	3169	HumanHap370Duo
				
Validation	SEARCH and RBCS	ER-negative cases	1011	Taqman
		Controls	7604	Taqman

ER, estrogen receptor; RBCS, Rotterdam Breast Cancer Study; SEARCH, Study of Epidemiology and Risk factors in Cancer Heredity.

Briefly, the Swedish sample set included subjects who were drawn from a parent population-based case control study of postmenopausal breast cancer which has been described elsewhere [24,25]. Case subjects were women born in Sweden who were 50 to 74 years of age at diagnosis and diagnosed with breast cancer between October 1993 and March 1995. A total of 803 individuals diagnosed with invasive breast cancer and with available blood samples were selected for GWAS genotyping in an independent GWAS looking at overall breast cancer risk [26]. Of these women, 153 individuals were diagnosed with the ER-negative disease and were included in the present study. In addition, a total of 1,414 Swedish controls were included from the parent study and an additional Epidemiological Investigation of Rheumatoid Arthritis (EIRA) study [27].

The Finnish breast cancer study population consists of two series of unselected breast cancer patients and additional familial cases ascertained at the Helsinki University Central Hospital. The first series of patients was collected in 1997 to 1998 and 2000 and covers 79% of all consecutive, newly diagnosed cases during the collection periods [28,29]. The second series, containing newly diagnosed patients, was collected in 2001 to 2004 and covers 87% of all such patients treated at the hospital during the collection period [30]. The collection of additional familial cases has been described previously [31]. We genotyped a total of 782 breast cancer cases in an independent GWAS for overall breast cancer risk [26], of which 226 ER-negative cases were used in the present study. An additional 238 Finnish ER-negative cases were also genotyped for this study, using a different platform. Of these 464 women with ER-negative breast cancer, 207 were sporadic and 257 were familial breast cancer cases. Population control data were obtained from the Finnish Genome Centre on 3,169 healthy population controls described in [32-35].

SEARCH is a population-based case-control study comprising 7,093 cases identified through the East Anglian Cancer Registry: prevalent cases diagnosed age <55 from 1991 to 1996 and alive when the study started in 1996, and incident cases diagnosed <70 diagnosed after 1996. Controls (*N *= 8,096) were selected from the EPIC-Norfolk cohort study, a population-based cohort study of diet and health based in the same geographical region as SEARCH, together with additional SEARCH controls recruited through general practices in East Anglian region.

RBCS is a hospital-based case-control study comprising 799 cases characterized as familial breast cancer patients selected from the Rotterdam Family Cancer Clinic at the Erasmus Medical Center, of which 141 are ER-negative. Controls (*N *= 801) were spouses or mutation-negative siblings of heterozygous Cystic Fibrosis mutation carriers selected from the Department of Clinical Genetics at the Erasmus Medical Center. Both cases and controls were recruited between 1994 and 2006.

### Genotyping and quality control filters

Genotyping for all samples was performed according to the Illumina Infinium 2 assay manual (Illumina, San Diego, CA, USA), as described previously [36]. The genotyping platforms used for this study are listed in Table 1. Apart from the 3,170 Finnish controls which were genotyped on the HumanHap370Duo assay as described previously [32,34], genotyping for all other Finnish and Swedish samples was performed at the Genome Institute of Singapore.

Each dataset was filtered to remove individuals with >10% missing genotypes, and SNPs with >10% missing data, or minor allele frequency (MAF) <0.03, or not in Hardy-Weinberg equilibrium (HWE) (*P *< 0.05/number of SNPs after quality control) and individual samples with evidence of possible DNA contamination, common ancestry or cryptic family relationships. Quality control was carried out using the software Plink [37]. To account for population outliers and correct for differential ancestry between cases and controls that may exist in the dataset after familial outlier removal, a principal component (PC) analysis was conducted using the EIGENSTRAT software (Broad Institute, Boston, MA, USA) [38].

A total of 617 ER-negative cases and 4,583 controls passed the quality control for samples. The 285,984 SNPs that passed quality control filters in all sample sets were merged into a single file for analysis.

The five most strongly associated SNPs in the combined analysis, which had effects in the same direction for both studies in the discovery stage (Swedish and Finnish) were forwarded for validation in SEARCH and RBCS. Genotyping in SEARCH and RBCS was performed by 5'exonuclease assay (Taqman) using the ABI Prism 7900HT sequence detection system (Applied Biosystems, Foster City, CA, USA) according to the manufacturer's instructions.

All SNP chromosomal positions were based on NCBI Build 36.

### Statistical analysis

**Figure 1:**
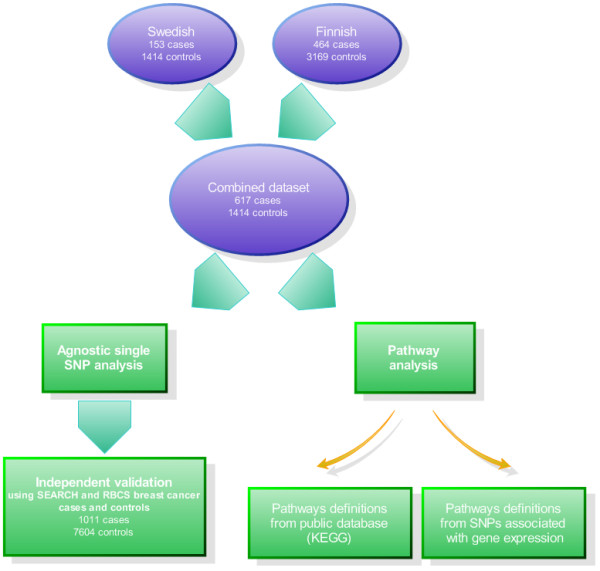
**Schematic diagram of analytical strategies for agnostic single marker association analysis and pathway analysis**.

Figure 1 gives a broad overview of the analytical strategy for the single marker association analysis and pathway analysis.

#### Single marker association analysis

Logistic regression models with genotype coded 0, 1, 2 and treated as a continuous covariate (one at a time), were fitted for each SNP that passed quality control. An additive genetic effect on the logit scale was assumed to characterize the associations. Separate analyses were performed for the Swedish and Finnish datasets as well as a combined analysis.

In the combined analysis, the final model included as covariates the SNP genotype, an indicator variable specifying country (Sweden and Finland), and interaction effects of Eigen values of PCs × country specified in such a way that country-specific PCs were implemented for the relevant subjects. Quantile-quantile plots were used to check for systematic genotyping error or bias due to unaccounted underlying population substructure. Manhattan plots were generated to summarize the -log transformed *P*-values of all SNPs examined.

#### Pathway analysis using discovery set (Swedish and Finnish samples)

Pathway analysis of the discovery GWAS dataset was conducted using the SNP ratio test (SRT) SRT was used to investigate the associations with breast cancer for 212 pathways and their genes (approximately 4,700) taken from the Kyoto Encyclopedia of Genes and Genomes (KEGG) database (05/12/08) [39].

To evaluate the association between regulatory SNPs-defined pathways and ER-negative breast cancer, we used the downloadable database from mRNA by SNP Browser [40] to map SNPs, which are significantly associated with gene expression on a genome-wide level (LOD >6), to genes. In total, 7,698 SNPs were mapped to 3,740 probes with a LOD score >6. These 3,740 probes could be mapped to 2,070 genes, and out of these, 554 genes, regulated by 1,720 SNPs, were annotated as belonging to one or several of the 182 KEGG pathways.

Among five regulatory SNP-defined pathways found to be significantly associated with ER-negative breast cancer, four belonged to the pathway class "cancer". To evaluate if the abundance of small *P*-values from regulatory SNPs involved in cancer-related pathways was statistically significant as a whole, we also assessed the departure of the distribution of the trend test statistics from the null distribution, assuming that none of the SNPs was associated with ER-negative breast cancer as an outcome. For this purpose, we performed the "admixture maximum likelihood" test described by Tyrer *et al. *[41] to obtain a global *P*-value for 165 unique SNPs from 15 cancer-related pathways (hsa052*) curated in the KEGG database.

#### Analysis of shared polygenic variation between ER-negative and ER-positive breast cancer subtypes

**Figure 2:**
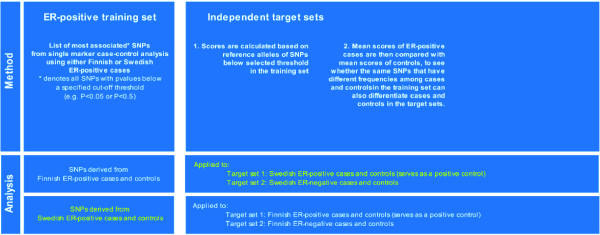
**Summary of scoring procedure for assessment of common polygenic variation**.

We assessed the polygenic component of breast cancer risk using a procedure for creating sample scores which has been described elsewhere [42]. Briefly, ER-positive breast cancer cases and healthy controls from either the Finnish or Swedish study were used as a "training set" to derive a list of SNPs used for scoring in two "target sets", consisting of either ER-positive breast cancer cases and healthy controls or ER-negative breast cancer cases and healthy controls in the other population. Figure 2 gives a broad overview of the analytical strategy for assessing common polygenic variation.

The polygenic score for each individual was calculated by summing the number of score alleles weighed by the log of their odds ratio from the training sample, across all SNPs included in the score. SNPs were included in the score if they achieved a *P*-value less than a particular threshold in the training sample. The "---score" function in Plink [37] was used to calculate scores. To capture association signals with very small effects in the calculation of the polygenic component of the disease, we used non-stringent significance thresholds (*P *< 0.01, *P *< 0.05, *P *< 0.10, *P *< 0.20, *P *< 0.30, *P *< 0.40 and *P *< 0.50). Scores were calculated for the seven *P*-value thresholds.

The extent of shared polygenic variation between ER-positive breast cancers in the training sample and ER-positive and ER-negative breast cancers in the corresponding target samples was assessed by fitting logistic regression models to disease state, as a function of score, in the target samples. Regression models, adjusted for the number of non-missing genotypes, were fitted to assess the differences in the extent of shared polygenic variation (scores) between the ER-positive and ER-negative target samples in case-only analyses.

PLINK (v1.06) [37], SNP Ratio Test [19], R (v2.8.0) [43], Quanto [44], AML [41], Qlikview (v8.5) [45], HaploView [46] and LocusZoom [47] were used for data management, quality control, statistical analyses, and graphics. All reported tests are two-sided.

## Results

In this study, we tested the association of 285,984 loci with ER-negative breast cancer in two independent populations consisting of a total of 617 cases and 4,583 controls. It appears that the overall population substructure was adequately accounted for, since a systematic deviation from the expected distribution was not observed in the quantile-quantile plot (Supplementary Figures 2, 3 and 4 in Additional file 2). Quantile-quantile plots generated from the analyses of individual datasets showed that there was no within-study systematic error arising from the use of non-matched population controls or genotyping at different facilities (Supplementary Figures 2 and 3 in Additional file 2). Genotype cluster plots were examined for SNPs with *P *< 10^-5^. Manual reclustering was performed for six SNPs with poor genotype cluster plots. SNPs rs4660646 and rs2462692 were omitted from further analysis as they could not be reclustered. SNPs rs4549482, rs1984492, rs1389545 and rs3748648 were not found to be strongly associated with ER-negative breast cancer after reclustering (Table S1 in Additional file 3).

**Figure 3:**
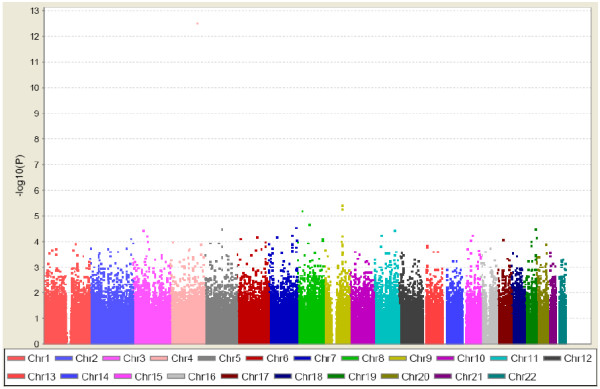
**Genome-wide *P*-values (-log_10_P) of the logistic regression analysis plotted against chromosomal position**.

**Figure 4:**
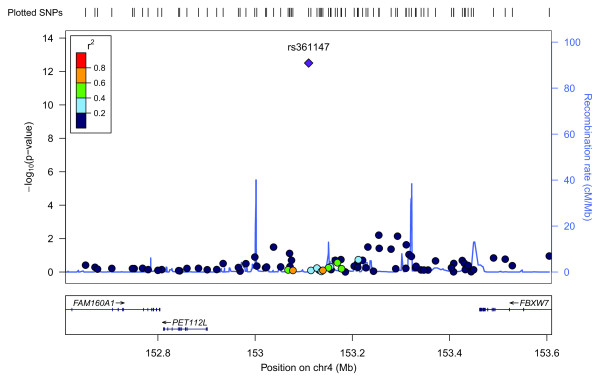
**Plot of regional association signals for rs361147 forwarded for validation**.

**Figure 5:**
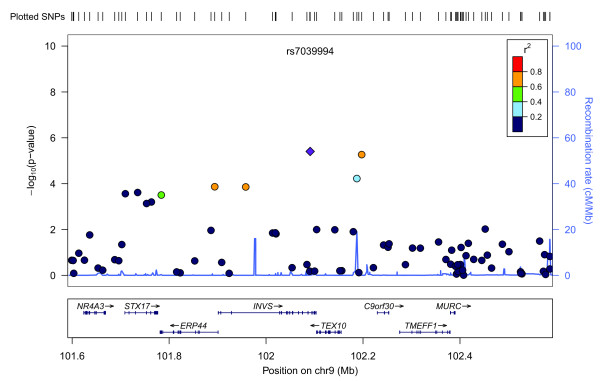
**Plot of regional association signals for rs7039994 forwarded for validation**.

**Figure 6:**
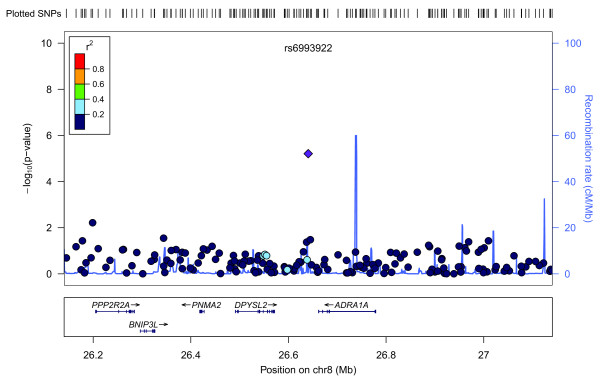
**Plot of regional association signals for rs6993922 forwarded for validation**.

**Figure 7:**
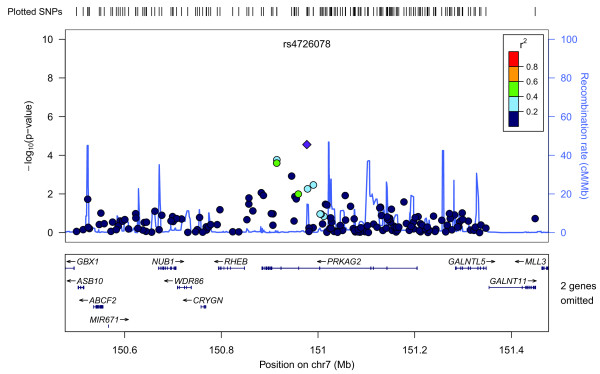
**Plot of regional association signals for rs4726078 forwarded for validation**.

**Figure 8:**
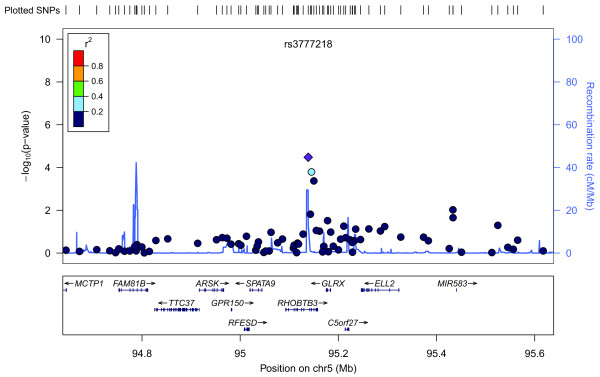
**Plot of regional association signals for rs3777218 forwarded for validation**.

Figure 3 shows a Manhattan plot summarizing the -log-transformed *P*-values of 285,984 SNPs analyzed in this study. In a combined analysis of individuals of Swedish and Finnish backgrounds, the strongest association with ER-negative breast cancer below the threshold for genome-wide significance was for a locus marked by rs361147 on chromosome 4 (*P *trend = 3.13 × 10^-13^; OR _per allele _= 0.60) (Table S2 in Additional file 3). This was the only SNP to achieve statistical significance at the genome-wide level (α = 5 × 10^-8^). Overall, no significant signal peak was identified in this study (Figures 4, 5, 6, 7, 8).

Nevertheless, we selected five SNPs to be validated in a combined dataset of two independent studies (Table S2 in Additional file 3). SNPs rs7039994 and rs12000794, located 106310 base pairs away from each other on chromosome 9, were found to be in high LD (r2 = 0.797; D' = 0.952). The former was kept and validated in the SEARCH dataset as its associated *P*-value was smaller and it was in closer proximity to coding regions (downstream of *INVS*|*TEX10*). SNP rs3777218 was selected over rs11882068 due to a better regional signal peak. Other SNPs selected for validation included rs361147 as mentioned above, rs6993922, rs4726078 (within transcript of *PRKAG2*), and rs3777218 (within transcript of *RHOBTB3*). Of the five SNPs forwarded for validation, rs4726078 could not be designed and was replaced by rs10952315 (r2 = 0.977 in Centre d'Etude du Polymorphisme Humain (CEPH) from Utah (CEU) HapMap samples). None of the SNPs was significantly associated at the 5% level in the second stage. The smallest *P*-value obtained was for the surrogate rs10952315 (OR 1.02; 95% CI: 0.93 to 1.13).

To analyze our GWAS data in a pathway context we conducted a permutation-based analysis using the KEGG database. Pathways defined by SNPs located within transcript of genes that were found to be significantly associated with ER-negative breast cancer after 1,000 phenotype permutations at a threshold of *P*_α = 0.05 _< 0.05 (uncorrected) were: pentose and glucuronate interconversions (hsa00040) (*P *= 0.022), starch and sucrose metabolism (hsa00500) (*P *= 0.042), and gap junction (hsa04540) (*P *= 0.037) (Table 2).

**Table 2:** Top ranking pathways of genome-wide pathway analysis results using SNP ratio test (*P *< 0.1)

KEGG ID	Pathway name *Class*	No. of SNPs *P *< 0.05	No. of SNPs in pathway	Number of significantly associated SNPs with *P*				
					
				E-05	E-04	E-03	E-02	*P*
00040	Pentose and glucuronate interconversions *Metabolism; Carbohydrate Metabolism*	11	63	0	1	2	8	0.022
								
04540	Gap junction *Cellular Processes; Cell Communication*	95	1,366	1	0	16	78	0.037
								
00500	Starch and sucrose metabolism *Metabolism; Carbohydrate Metabolism*	22	237	0	2	4	16	0.042
								
00604	Glycosphingolipid biosynthesis ganglio series *Metabolism; Glycan Biosynthesis and Metabolism*	20	216	0	0	4	16	0.051
								
00230	Purine metabolism *Metabolism; Nucleotide Metabolism*	106	1,618	1	2	16	87	0.054
								
04130	SNARE interactions in vesicular transport *Genetic Information Processing; Folding, Sorting and Degradation*	19	206	0	4	1	14	0.060
								
03022	Basal transcription factors *Genetic Information Processing; Transcription*	11	105	0	0	4	7	0.062
								
04910	Insulin signaling pathway *Cellular Processes; Endocrine System*	61	889	2	6	9	44	0.071
								
04350	TGF-beta signaling pathway *Environmental Information Processing; Signal Transduction*	43	586	0	1	9	33	0.077
								
04330	Notch signaling pathway *Environmental Information Processing; Signal Transduction*	25	321	0	0	4	21	0.087
								
04614	Renin-angiotensin system *Cellular Processes; Endocrine System*	8	78	0	0	1	7	0.092

KEGG ID, Kyoto Encyclopedia of Genes and Genomes pathway identifier (hsa*); P, P-value of permutation test; SNP, single nucleotide polymorphism

In addition, we limited the analysis to pathway definitions involving only known regulatory SNPs [48]. The GWAS SNPs were first mapped to genes, and then subsequently to KEGG pathways based on publicly available gene regulatory data from lymphoblastoid cells [48]. Only genes with regulatory functions significant on a genome-wide significant level were selected, resulting in 1,720 SNPs regulating members of 182 KEGG pathways being used in our analysis. Pathways that were found to be significant by SRT after 1,000 phenotype permutations at a threshold of *P*_α = 0.05 _< 0.05 were: long-term potentiation (hsa04720), glioma (hsa05214), non-small cell lung cancer (hsa05223), pancreatic cancer (hsa05212), and prostate cancer (hsa5215) (Table 3). The focal adhesion pathway (hsa04510) was found to be marginally significant (*P*_α = 0.05 _= 0.052). Two pathways each tagged by only a single SNP, glyoxylate and dicarboxylate metabolism (hsa00630) and glycosphingolipid biosynthesis - ganglio series (hsa00604), were removed from the evaluation of the final results.

**Table 3:** Top ranking pathways of genome-wide pathway analysis using regulatory SNPs

		*P*-value distribution of SNPs				
				
Pathway name (KEGG ID) *Class*	SRT P	***P* < 0.01**	0.01 ≤ *P *< 0.05	0.05 ≤ *P *< 0.1	N	*P *of most significant SNP in pathway
Glioma (hsa05214) *Cancers*	0.0394	1	5	4	26	0.0028
Long-term potentiation (hsa04720) *Nervous System*	0.0394	0	3	2	16	0.0314
Non-small cell lung cancer (hsa05223) *Cancers*	0.0394	1	5	3	24	0.0028
Pancreatic cancer (hsa05212) *Cancers*	0.0413	2	5	3	33	0.0028
Prostate cancer (hsa05215) *Cancers*	0.0488	3	3	6	32	0.0003
Focal adhesion (hsa04510) *Cell Communication*	0.0525	1	7	9	71	0.0028
Chemokine signaling pathway (hsa04062) *Immune System*	0.0582	1	8	7	72	0.0080
Pathways in cancer (hsa05200) *Cancers*	0.0582	2	12	15	151	0.0028
Melanogenesis (hsa04916) *Endocrine System*	0.0657	2	2	2	26	0.0003
B cell receptor signaling pathway (hsa04662) *Immune System*	0.0713	0	5	3	29	0.0314
GnRH signaling pathway (hsa04912) *Endocrine System*	0.0732	0	6	6	46	0.0115
Fc epsilon RI signaling pathway (hsa04664) *Immune System*	0.0769	0	6	6	33	0.0314
VEGF signaling pathway (hsa04370) *Signal Transduction*	0.0769	0	3	0	17	0.0115
ErbB signaling pathway (hsa04012) *Signal Transduction*	0.0788	0	5	5	25	0.0314
Acute myeloid leukemia (hsa05221) *Cancers*	0.0957	1	3	3	25	0.0028
Gap junction (hsa04540) *Cell Communication*	0.0976	0	5	3	42	0.0314

KEGG ID, Kyoto Encyclopedia of Genes and Genomes pathway identifier; P, P-value of association test in the genome-wide study; SNP, single nucleotide polymorphism; SRT P, P-value of permutation test for pathway tested

**Figure 9:**
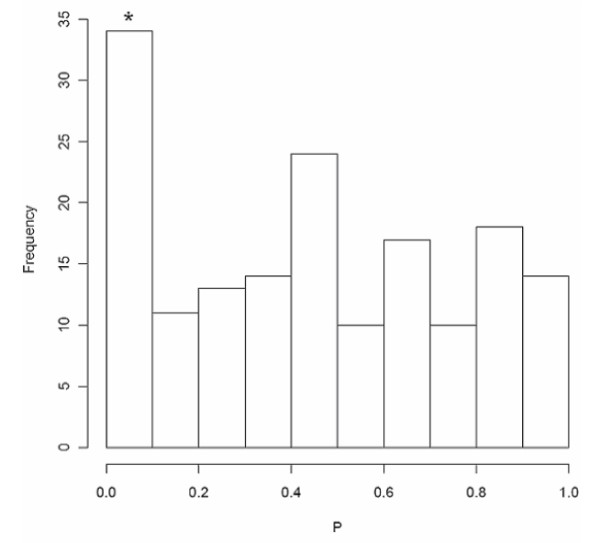
Distribution of *P*-values of regulatory SNPs within KEGG cancer pathways (pathway identifiers beginning with hsa052*). *Global *P*-values of cancer-related regulatory SNPs with *P *< 0.05 in the genome-wide association analysis using the admixture maximum likelihood test (5,000 permutations) are 0.0028 (unadjusted), and 0.052 (with adjustments made to correct for population stratification).

Regulatory SNPs involved in pathways associated with cancer (hsa052*) appeared to be overrepresented by small *P*-values (Figure 9). To evaluate if the combined effect of these signals was statistically significant as a whole, we next carried out a global test of significance for all unique SNPs in the cancer pathways. The AML analysis performed using an algorithm developed by Tyrer *et al. *[41], yielded *P*-values (α = 0.05) of 0.0028 (crude) and 0.052 (adjusted for population stratification).

**Figure 10:**
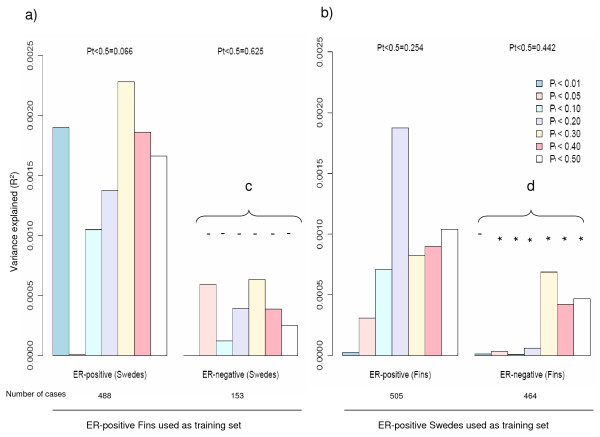
**Proportion of shared polygenic component between breast cancer estrogen receptor subtypes**. Proportion of shared polygenic component between ER-positive and ER-negative target samples, with respect to their corresponding ER-positive training samples. Pt denotes *P*-value cut-off in training sample. **a) **Test for association between polygenic score and disease status (ER-positive/ER-negative) in the Swedish data, when all SNPs with *P *< 0.5 in the Finnish training set were considered. **b) **Test for association between polygenic score and disease status (ER-positive/ER-negative) in the Finnish data, when all SNPs with *P *< 0.5 in the Swedish training set were considered. **c) **Significance test for difference in scores (Finnish ER-positive breast cancers derived) between Swedish ER-negative and ER-positive breast cancers, adjusted for number of non-missing genotypes. Significance codes: '- ' 0.1 <*P *< 1 (that is, not significant). **d) **Significance test for difference in scores (Swedish ER-positive breast cancers derived) between Finnish ER-negative and ER-positive breast cancers, adjusted for number of non-missing genotypes. Significance codes: '*' 0.01 <*P *< 0.05.

Figure 10 shows the results of analyses aimed at assessing the shared polygenic component between ER-positive and ER-negative breast cancer. Estimates of variance explained in datasets indicate how important the polygenic component of ER-positive disease is in explaining the overall occurrence of ER-positive and ER-negative diseases. The proportion of variance explained for all categories of *P*-value cut-offs, with the exception of *P *< 0.05 in the Swedish ER-positive target sample, was higher in the ER-positive target datasets than the ER-negative target datasets.

We test for association between polygenic score and disease status (ER-positive vs controls/ER-negative vs controls) in the target data, when seven groups of SNPs with different *P*-values thresholds in the training sets were considered (Figure 10a, b). Due possibly to limited statistical power (Table S3 in Additional file 3), even at the least stringent *P*-value threshold (*P *< 0.50), the ER-positive and ER-negative breast cancer target case-control datasets failed to provide statistically significant evidence of a polygenic component for ER-positive cancer, or evidence of a polygenic component shared between the two cancers, when training was based on the ER-positive training case-control datasets (Figure 10a, b). Nevertheless, when we relaxed the *P*-value cut-off in the training dataset to 0.5, the Swedish ER-positive breast cancer target dataset showed borderline significance for a shared polygenic component with ER-positive breast cancer, based on the Finnish ER-positive training dataset (Figure 10a, *P *= 0.066).

In a separate case-only analysis, we performed a significance test for difference in scores between ER-positive and ER-negative breast cancer cases in the target data. Significant results show that ER-positive and ER-negative breast cancers are not identical diseases (genetically at polygenic level) (Figures 10c, d). The difference in scores between ER-positive and ER-negative samples was found to be statistically significant for all categories of *P*-value cut-offs in the Finnish target case-only samples, with the exception of the most associated SNPs (Figure 10d).

## Discussion

Little is known about the genetic predisposition to estrogen receptor-negative breast cancer. This subtype is characterized by lower age of onset, a more aggressive disease and low or no response to selective estrogen receptor modulators or aromatase inhibitors. We have examined our GWAS data on two different levels: single marker and pathway. We also provided evidence that breast cancer is a heterogeneous disease with a polygenic nature, with significant differences between the polygenic component between ER-positive and ER-negative breast cancers. This emphasizes the importance of looking at ER-negative breast cancer separately as a unique breast cancer phenotype.

Overall, no significant signal peak was identified in this study (Figures 4, 5, 6, 7, 8). Only one SNP (rs361147) was found to achieve genome-wide significance after correction for multiple testing in the single marker analysis. However, the other loci exhibiting strong associations were interesting for reasons of biological significance, and were considered to merit further research. The associated region on 9q31.1 tagged by rs7039994 contains two known genes, *TEX10 *(testis expressed sequence 10) and *INVS *(inversin). No function has been ascribed to *TEX10*. *INVS *is reported to function as a molecular switch between different Wnt signalling pathways [49] and is also pivotal in the establishment of the left-right axis. The *RHOBTB3 *gene, harbouring SNP rs3777218, was identified as a putative breast cancer anti-estrogen resistance gene [50]. However, none of these single markers most strongly associated with ER-negative breast cancer could be replicated in a larger, independent sample made up of two independent studies (Table 1)

To maximize the information obtained from the GWAS scan, we conducted a permutation-based pathway analysis using the KEGG database to capture the joint actions of multiple SNPs with modest effects. In the analysis using default SRT pathway definition files comprising within-transcript SNPs, metabolic pathways involving pentose and glucuronate interconversions (hsa00040) (*P *= 0.022) as well as starch and sucrose metabolism (hsa00500) (*P *= 0.042) were found to be nominally significantly related to the risk of developing ER-negative breast cancer (Table 2). Estrogen-induced breast cancer cell proliferation is often accompanied by an increase in intracellular metabolic activity, resulting in a higher growth rate. The pentose phosphate pathway, which works in tight conjunction with the pentose and glucuronate interconversions and starch and sucrose metabolism pathways, has recently been suggested to be essential for estrogen-dependent cell proliferation [51]. Several pathways that were found to be marginally significant (*P *< 0.1) have been suggested to have potential roles in ER-negative breast cancer, namely, the TGF-beta signalling pathway [52], the renin-angiotensin system [53], and the Notch signalling pathway [54]. In addition, the insulin signalling pathway has been the focus of targeted therapy for breast cancer [55], and the purine metabolism pathway is also closely related to the pentose phosphate pathway described earlier.

Nevertheless, there is neither a precise biological definition of a pathway, nor a "standard" method to map SNPs to genes, and then genes to pathways. Pathway analyses of GWAS of common diseases have mostly based SNP-to-gene mappings on the chromosomal position of the SNP, whether it occurs within transcript of a certain gene [19,56]. However, it may be more meaningful to map SNPs that are associated with the expression of a gene to the gene. To elucidate pathways with more biological relevance, we further conducted pathway analysis based on a subset of SNPs with known regulatory functions. Recent studies have observed that whereas stronger effects overlap between different tissues, weak effects on gene regulation are tissue-specific [57,58]. Since we utilized data on gene regulation from lymphoblasts, we decided to restrict our dataset to only genes regulated on a genome-wide significant level (LOD >6). This minimized the bias of tissue-specific gene regulation, but at the same time, limited us to only a fraction of all possible SNPs genotyped within our GWAS, thus reducing the power of the analysis.

In spite of the limitations, four of the five significantly associated pathways (*P *< 0.05) in our analysis were found to be annotated as cancer pathways in KEGG (glioma (hsa05214), non-small cell lung cancer (hsa05223), pancreatic cancer (hsa05212), and prostate cancer (hsa05215) (Table 3)), hence confirming the validity of the choice of this subset of regulatory SNPs in pathway definition. In addition, a global test of the SNPs defining the cancer pathways found the aggregate effect to be approaching statistical significance (*P*_α = 0.05 _= 0.052). Due to the large number of markers evaluated in a genome-wide scan, signals with small effects and modestly significant *P*-values are likely to be dismissed after the correction of multiple testing. The implementation of a pathway analysis thus serves as a complementation between a hypothesis-driven (prior knowledge of biological pathways) and a hypothesis-free (genome-wide scan) approach to highlight certain markers, such as those found in the cancer pathways, worthy of further study that would not have been examined otherwise. The lack of a concordance between the results of pathway analyses using two different SNP-to-gene mapping approaches emphasizes the need to put in more consideration in choosing appropriate pathway definitions. An excess of small *P*-values found for SNPs associated with gene expression involved in cancer-related pathways suggests that the SNP-gene mapping via association with gene expression approach is superior to the SNP-gene mapping by location within a transcript approach, and should be explored in greater detail.

Limitations of this study include an overall lack of statistical power, especially for the single marker analysis, and the existence of further heterogeneity among ER-negative tumours. Although genome-wide pathway-based analysis is an interesting approach, a main limitation is that the associations observed in this study are only nominally significant, and would not be significant after correction for multiple testing. However, as many pathways have SNPs in common with other pathways, the stringent significance thresholds of traditional multiple testing correction methods are potentially over-conservative. There is also indirect evidence that corroborates our pathway findings. Gene expression studies have found pathways related to the renin-angiotensin system and focal adhesion to be significantly associated with prognosis of breast cancer [59]. Others have also reported pathways highlighted in our study, which are involved in pentose and glucuronate interconversions, gap junction, TGF-beta signalling, rennin-angiotensin system, B cell receptor signalling, Fc epsilon RI signalling, VEGF signalling, ErbB signalling, and focal adhesion, to be significantly associated with the breast cancer phenotype [59,60]. Although replication of the pathway results in independent studies would be needed to confirm the associations, the substantial additional sample collection and genotyping required are beyond the scope of this publication.

Although breast cancer has been classified into ER-positive and ER-negative breast cancers, and these two breast cancer subtypes have been documented to show different gene expression patterns, GWAS scans on breast cancer have always been performed on either overall breast cancer (ER-positive, ER-negative and unknown) or ER-positive breast cancer specific risks. In this study, we found evidence to suggest that ER-negative breast cancers only share a fraction of the polygenic component of the disease with ER-positive breast cancers, implying that ER-negative breast cancer should be examined as a distinct breast cancer phenotype. Although the difference between the polygenic components of ER-positive and ER-negative breast cancers was found only to be significant in the Finnish training samples, we observed similar differences for all seven *P*-value thresholds in the Swedish training samples. However, due to the smaller number of Swedish ER-negative cases (*N *= 153, approximately 33% of Finnish ER-negative cases), we had less power to detect significant heterogeneity between the two subtypes in the Swedish target samples.

## Conclusions

Given the clinical importance of the ER-negative phenotype and the likelihood that the relative genetic effect sizes are small, greater sample sizes and further studies are required to further the knowledge on ER-negative breast cancers. Identification of factors for a predisposition to ER-negative tumours opens the way for understanding the underlying etiology of the disease, and may ultimately result in improvements in prevention, early detection and specific treatment for this tumour subtype. We used a novel approach to pathway analysis, showing that established cancer pathways could be regulated by common variants associated to ER-negative breast cancer. We also provided molecular genetic evidence which suggests that ER-negative breast cancer is a distinct breast cancer subtype that merits independent analyses. In view of the biological relevance of the pathways identified, a genome-wide pathway approach deserves merit, and has good potential in pointing out directions for future research for ER-negative breast cancers.

## Abbreviations

Λ: genomic control inflation factor; AML: admixture maximum likelihood; EIRA: Epidemiological Investigation of Rheumatoid Arthritis; ER: estrogen receptor; GWAS: genome-wide association study; HWE: Hardy-Weinberg equilibrium; KEGG: Kyoto Encyclopedia of Genes and Genomes; LD: linkage disequilibrium; MAF: minor allele frequency; NCBI: National Center for Biotechnology Information; PC: principal component; PCA: principal component analysis; RBCS: Rotterdam Breast Cancer Study; SEARCH: Study of Epidemiology and Risk factors in Cancer Heredity; SNP: single nucleotide polymorphism; SRT: SNP ratio test.

## Competing interests

The authors declare that they have no competing interests.

## Authors' contributions

JLi, KH, HN, JLiu, KC, and PH conceived and designed the experiments. JLi, KH, HD, UH, TH, AI, HQL, GHKT, AT and GR analyzed the data. KA, CB, PDPP, AMD, DA, MJH, AH, RAO, LA, AP, LPP, JK, MD, DFE, HN, JLiu, KC and PH contributed reagents/materials/analysis tools. JLi, KH, HD, GR, UH, TH, KA, CB, PDPP, AMD, DA, MJH, AH, RAO, LA, AP, LPP, AI, HQL, GHKT, AT, JK, MD, DFE, HN, JLiu, KC and PH wrote the paper.

## Supplementary Material

Additional file 1 **Supplementary Methods**. Full methods accompanying this manuscript. Click here for file

Additional file 2 **Supplementary figures**. Supplementary Figure 1. Scree plot of log-transformed Eigen values. Vertical dashed lines indicate three and five PCs taken to correct for population stratification within the Swedish and Finnish populations respectively. Supplementary Figure 2. Quantile-quantile plot for 285,984 SNP trend tests, adjusted for population stratification using three principal components (Swedish subjects only). Genomic control inflation factor (λ) = 1.0140. Supplementary Figure 3. Quantile-quantile plot for 285,984 SNP trend tests, adjusted for population stratification using five principal components (Finnish subjects only). Genomic control inflation factor (λ) = 1.0137. Supplementary Figure 4. Quantile-quantile plot for 285,984 SNP trend tests, adjusted for population stratification (combined analysis of Swedish and Finnish subjects). Genomic control inflation factor (λ) = 1.0218. Click here for file

Additional file 3 **Supplementary tables**. Table S1. Association analysis results of reclustered SNPs. Table S2. Association results of top hits in the combined analysis, with corresponding MAF, ORs and P values within the Swedish and Finnish populations. * denotes the five SNPs selected for validation in SEARCH and RBCS. Table S3. Power to detect single marker effects in genome-wide association study. Click here for file
