# Supplementary material for: A genome-wide association scan on estrogen receptor-negative breast cancer
Source: Breast Cancer Res. 2010 Nov 9;12(6):R93. doi: 10.1186/bcr2772 (PMC3046434; doi:10.1186/bcr2772)
Supplement: Additional file 1 — Supplementary Methods. Full methods accompanying this manuscript. [file bcr2772-S1.pdf]

Additional File 1: Full methods

Materials and Methods 2
Study populations used in the discovery stage 2
Genotyping and quality control filters 3
Statistical analysis 5
Single marker associaton analysis 6
Validation of single marker association analysis in independent samples 6
Pathway analysis using discovery set (Swedish and Finnish samples) 7
Analysis of shared polygenic variation between ER-negative and ER-positive breast cancer subtypes 9

Materials and Methods

Study populations used in the discovery stage

Table 1 in Additional File 2 summarizes the demographics of cases and controls used in this study. The discovery stage consists of cases and controls from Finland and Sweden.

The Swedish sample set included subjects who were drawn from a parent population-based case control study of postmenopausal breast cancer which has been described elsewhere [24, 25]. Case subjects were women born in Sweden who were 50-74 years of age at diagnosis and diagnosed with breast cancer between October 1993 and March 1995. A total of 803 individuals diagnosed with invasive breast cancer and with available blood samples were selected for GWAS genotyping in an independent GWAS looking at overall breast cancer risk [26]. Of these women, 154 individuals were diagnosed with the ER-negative disease and were included in the present study. In addition, 764 controls, frequency matched to the age distribution of the cases within 5-year age intervals, were randomly chosen from the parent study. An additional 659 cancer-free Swedish controls aged between 18 and 70 years were obtained from the Epidemiological Investigation of Rheumatoid Arthritis (EIRA) study [27], primarily in order to improve the resolution of adjustment for population stratification in the association analyses.

The Finnish breast cancer study population consists of two series of unselected breast cancer patients and additional familial cases ascertained at the Helsinki University Central Hospital. The first series of patients was collected in 1997-1998 and

2000 and covers 79% of all consecutive, newly diagnosed cases during the collection periods [28, 29]. The second series, containing newly diagnosed patients, was collected in 2001 – 2004 and covers 87% of all such patients treated at the hospital during the collection period [30]. The collection of additional familial cases has been described previously [31]. We genotyped a total of 782 breast cancer cases in an independent GWAS for overall breast cancer risk [26], of which 226 ER-negative cases were used in the present study. An additional 238 Finnish ER-negative cases were also genotyped for this study, using a different platform. Of these 464 women with ER-negative breast cancer, 207 were sporadic and 257 were familial breast cancer cases. Population control data was obtained from the Finnish Genome Centre on 3,170 healthy population controls described in [32-35].

For all populations, blood samples were obtained from individuals according to protocols and informed-consent procedures approved by institutional review boards.

Genotyping and quality control filters

Genotyping for all samples was performed according to the Illumina Infinium 2 assay manual (Illumina, San Diego), as described previously [36]. The genotyping platforms used for this study are listed in Table 1 in Additional File 2. Apart from the 3,170 Finnish controls which were genotyped on the HumanHap370Duo assay as described previously [32, 34], genotyping for all other Finnish and Swedish samples was performed at the Genome Institute of Singapore.

Each dataset was filtered to remove individuals with >10% missing genotypes, and SNPs with >10% missing data, or minor allele frequency (MAF) < 0.03, or not in Hardy-Weinberg equilibrium (HWE) (P < 0.05/number of SNPs after quality control) and individual samples with evidence of possible DNA contamination, common ancestry or cryptic family relationships. Quality control was carried out using the software Plink [37]. Two individuals in the Finnish samples were found to have a full sibling relationship each other. The individual with the higher call rate was kept. Although the samples included in our study were derived predominantly from a European background, a small fraction of the study population may represent other ancestral backgrounds. To account for population outliers and correct for differential ancestry between cases and controls that may exist in the dataset after familial outlier removal, a principal component (PC) analysis was conducted using the EIGENSTRAT software [38]. One subject was removed from the Swedish breast cancer case study population, and nine were removed from the EIRA control study population. No PC analysis outliers were found for the Finnish study population. A total of 617 ER-negative cases and 4,583 controls passed the quality control for samples. The 285,984 SNPs that passed quality control filters in all sample sets were merged into a single file for analysis. As an additional quality control check, genotype cluster plots of the top SNPs (lowest p-values) from the discovery stage were inspected manually using Illumina Beadstudio 3.1 software (genotyping module) to confirm the genotype calling [61]. The five most strongly associated SNPs in the combined analysis, which had effects in the same direction for both studies (Swedish and Finnish) were forwarded for validation in independent studies. All SNP chromosomal positions were based on NCBI Build 36.

Eigenstrat-based principal component analysis (PCA) was used to summarize stratification and batch effects separately in the Swedish and Finnish datasets [38, 62]. A subset of linkage-disequilibrium (LD) thinned SNPs was selected such that all pair-wise associations had $r2<0.2$, as suggested by Patterson et al [62]. In addition, long-range regions of high LD, reported to potentially confound genome scans in admixed populations [63], were removed. After pruning, a total of 61,226 SNPs remained for PCA. The number of PCs to be used for adjustment was determined by plotting the log-transformed eigenvalues of each population and locating the position of the “elbow” on the Scree plot [64]. In our SNP association analyses of the full dataset of 285,984 SNPs, we adjusted for stratification using three and five PCs (obtained using the pruned dataset) in the Swedish and Finnish populations respectively (Supplementary Figure 1 in Additional File 3). The genomic control inflation factor λ was computed by taking the median of the distribution of the chi-square statistic from results of PC-adjusted logistic regression analyses of individual datasets, and dividing this median by the median of the corresponding (ideal) chi-square distribution (0.456) [65]. The genomic control inflation factors for the Swedish, Finnish and combined datasets were 1.0140, 1.0137, and 1.0218, respectively (Supplementary Figures 2-4 in Additional File 3) after correction for population stratification.

Statistical analysis

Figure 1 gives a broad overview of the analytical strategy for the single marker association analysis and pathway analysis.

Single marker associaton analysis

Logistic regression models with genotype coded 0, 1, 2 and treated as a continuous covariate (one at a time), were fitted for each SNP that passed quality control. An additive genetic effect on the logit scale was assumed to characterize the associations. Eigenvalues of PCs were included as covariates. Separate analyses were performed for the Swedish and Finnish datasets as well as a combined analysis. In the combined analysis, the final model included as covariates the SNP genotype, an indicator variable specifying country (Sweden and Finland), and interaction effects of eigenvalues of PCs × country specified in such a way that country-specific PCs were implemented for the relevant subjects. Quantile-quantile plots were used to check for systematic genotyping error or bias due to unaccounted underlying population substructure. Manhattan plots were generated to summarize the –log transformed P values of all SNPs examined. Pair-wise linkage disequilibrium (LD) was evaluated for the top SNPs that were observed to be located in the same chromosomal region using Plink [37].

Validation of single marker association analysis in independent samples

Five SNPs with the strongest association signals in the discovery data set were typed in two further studies: the Study of Epidemiology and Risk factors in Cancer Heredity (SEARCH) and Rotterdam Breast Cancer Study (RBCS), both previously described in Lesueur et al.[23] (1011 ER-negative cases, 7604 controls). SEARCH is a population-based case-control study comprising 7093 cases identified through the East Anglian Cancer Registry: prevalent cases diagnosed age <55 from 1991-1996 and alive when the study started in 1996, and incident cases diagnosed <70 diagnosed after 1996. Controls (N=8096) were selected from the EPIC-Norfolk cohort study, a population-

based cohort study of diet and health based in the same geographical region as SEARCH, together with additional SEARCH controls recruited through general practices in East Anglian region. Additional cases (N=799) and controls (N=801) from the RBCS were also genotyped. RBCS is a hospital-based case-control study comprising cases characterized as familial breast cancer patients selected from the Rotterdam Family Cancer Clinic at the Erasmus Medical Center. Controls were spouses or mutation-negative siblings of heterozygous Cystic Fibrosis mutation carriers selected from the Department of Clinical Genetics at the Erasmus Medical Center. Both cases and controls were recruited between 1994 and 2006. Genotyping in SEARCH and RBCS was performed by 5'exonuclease assay (Taqman) using the ABI Prism 7900HT sequence detection system according to the manufacturer's instructions. Primers and probes were supplied directly by Applied Biosystems as Assays-By-Design. Assays included at least two negative controls and 2-5% duplicates per plate.

Pathway analysis using discovery set (Swedish and Finnish samples)

Pathway analysis of the discovery GWAS dataset was conducted using the SNP ratio test (SRT) [19]. The same logistic regression models which were applied to the real dataset were applied to 1,000 datasets in which phenotypes were permuted, in order to obtain p-value estimates. SRT was used to investigate the associations with breast cancer for 212 pathways and their genes (~ 4,700) taken from the Kyoto Encyclopedia of Genes and Genomes (KEGG) database (05/12/08) [39]. SNP to gene mappings were obtained by parsing the dbSNP table b129_SNPContigLocusId_36_3.bcp. This includes SNPs <2kb 5' and <0.5kb 3' of a gene.

To evaluate the association between regulatory SNPs-defined pathways and ER-negative breast cancer, we used the downloadable database from mRNA by SNP Browser [40] to map SNPs, which are significantly associated with gene expression on a genome-wide level (LOD>6), to genes. Reported Affymetrix U133A 2.0 Plus Genechip Arrays probe id:s were converted to Entrez gene id:s using the November 2009 (release 30) annotation file provided by Affymetrix [66]. Entrez gene id:s were mapped to KEGG pathways using annotation files downloaded on 2009-12-10 [67]. In total, 7,698 SNPs were mapped to 3,740 probes with a LOD score > 6. These 3,740 probes could be mapped to 2,070 genes, and out of these, 554 genes, regulated by 1,720 SNPs, were annotated as belonging to one or several of the 182 KEGG pathways.

Among five regulatory SNP-defined pathways found to be significantly associated with ER-negative breast cancer, four belonged to the pathway class “cancer”. To evaluate if the abundance of small p-values from regulatory SNPs involved in cancer-related pathways was statistically significant as a whole, we also assessed the departure of the distribution of the trend test statistics from the null distribution, assuming that none of the SNPs was associated with ER-negative breast cancer as an outcome. For this purpose, we performed the “admixture maximum likelihood” test described by Tyrer et al. [41], selected because it has been previously shown to perform similarly to, or better than all other tests across a wide range of alternative hypotheses [41], to obtain a global p-value for 165 unique SNPs from 15 cancer-related pathways (hsa052*) curated in the KEGG database. Genomic control was used to correct for population stratification.

Analysis of shared polygenic variation between ER-negative and ER-positive breast cancer subtypes

We assessed the polygenic component of breast cancer risk using a procedure for creating sample scores which has been described elsewhere [42]. ER-positive breast cancers from our Finnish study (505 Finnish breast cancer cases), together with a subset of the available controls, were used in a training sample to create target sample scores. The full GWAS panel was further quality-controlled to retain SNPs with >99% genotyping success rate, and subsequently pruned to remove SNPs in strong linkage disequilibrium with other SNPs (based on a pairwise r2 threshold of 0.2, within a 200-SNP sliding window), to ensure the score represents the aggregate effect of a large number of independent SNPs. Figure 2 gives a broad overview of the analytical strategy for assessing common polygenic variation.

In the two target samples, Swedish ER-positive breast cancers (N=488) with a subset of the controls, and Swedish ER-negative breast cancers (N=153) with a corresponding subset of the controls, the polygenic score for each individual was calculated by summing the number of score alleles weighed by the log of their odds ratio from the training sample, across all SNPs included in the score. SNPs were included in the score if they achieved a p-value less than a particular threshold in the training sample. Where genotypic information was missing, imputation of mean scores was performed based on the target sample allele frequency. The same scoring procedure was carried out for two other target samples, Finnish ER-positive cases and corresponding controls, and Finnish ER-negative cases (N=464) and a subset of the remaining controls, using Swedish ER-positive cases (N=488) with corresponding controls as a training set. The --

score function in Plink [37] was used to calculate scores. To capture association signals with very small effects in the calculation of the polygenic component of the disease, we used non-stringent significance thresholds ($P<0.01$, $P<0.05$, $P<0.10$, $P<0.20$, $P<0.30$, $P<0.40$ and $P<0.50$). Scores were calculated for the seven p-value thresholds.

The extent of shared polygenic variation between ER-positive breast cancers in the training sample and ER-positive and ER-negative breast cancers in the corresponding target samples was assessed by fitting logistic regression models to disease state, as a function of score, in the target samples. The number of non-missing genotypes of all SNPs used to calculate the score was included as an adjustment covariate to control for potential differences in genotyping rate between cases and controls. Principal components were also included as adjustment covariates to correct for population stratification. The variance in disease state explained by the score (pseudo R2) was estimated as the difference in the deviances of a model including the score (and covariates) and a null model including only the covariates, divided by the deviance of the null model [68].

Regression models, adjusted for the number of non-missing genotypes, were fitted to assess the differences in the extent of shared polygenic variation (scores) between the ER-positive and ER-negative target samples in case-only analyses.

PLINK (v1.06) [37], SNP Ratio Test [19], R (v2.8.0) [43], Quanto [44], AML [41], Qlikview (v8.5) [45], HaploView [46] and LocusZoom [47] were used for data management, quality control, statistical analyses, and graphics. All reported tests are two-sided.
