# Supplementary material for: A genome-wide association scan on estrogen receptor-negative breast cancer
Source: Breast Cancer Res. 2010 Nov 9;12(6):R93. doi: 10.1186/bcr2772 (PMC3046434; doi:10.1186/bcr2772)

Supplementary Figure 1. Scree plot of log-transformed Eigenvalues. Vertical dashed lines indicate three and five PCs taken to correct for population stratification within the Swedish and Finnish populations respectively.

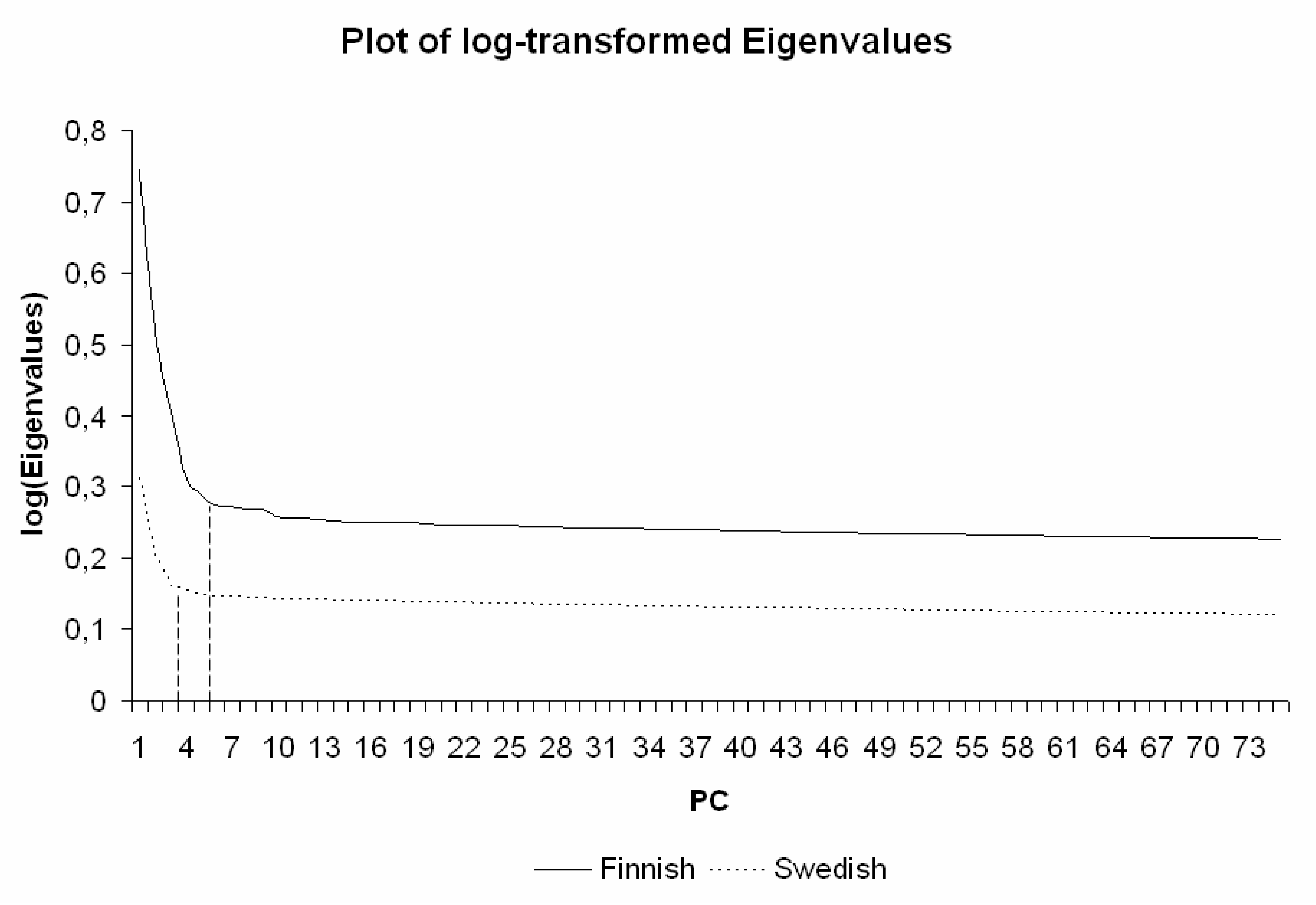

Supplementary Figure 2. Quantile-quantile plot for 285,984 SNP trend tests, adjusted for population stratification using three principal components (Swedish subjects only). Genomic control inflation factor (λ) = 1.0140.

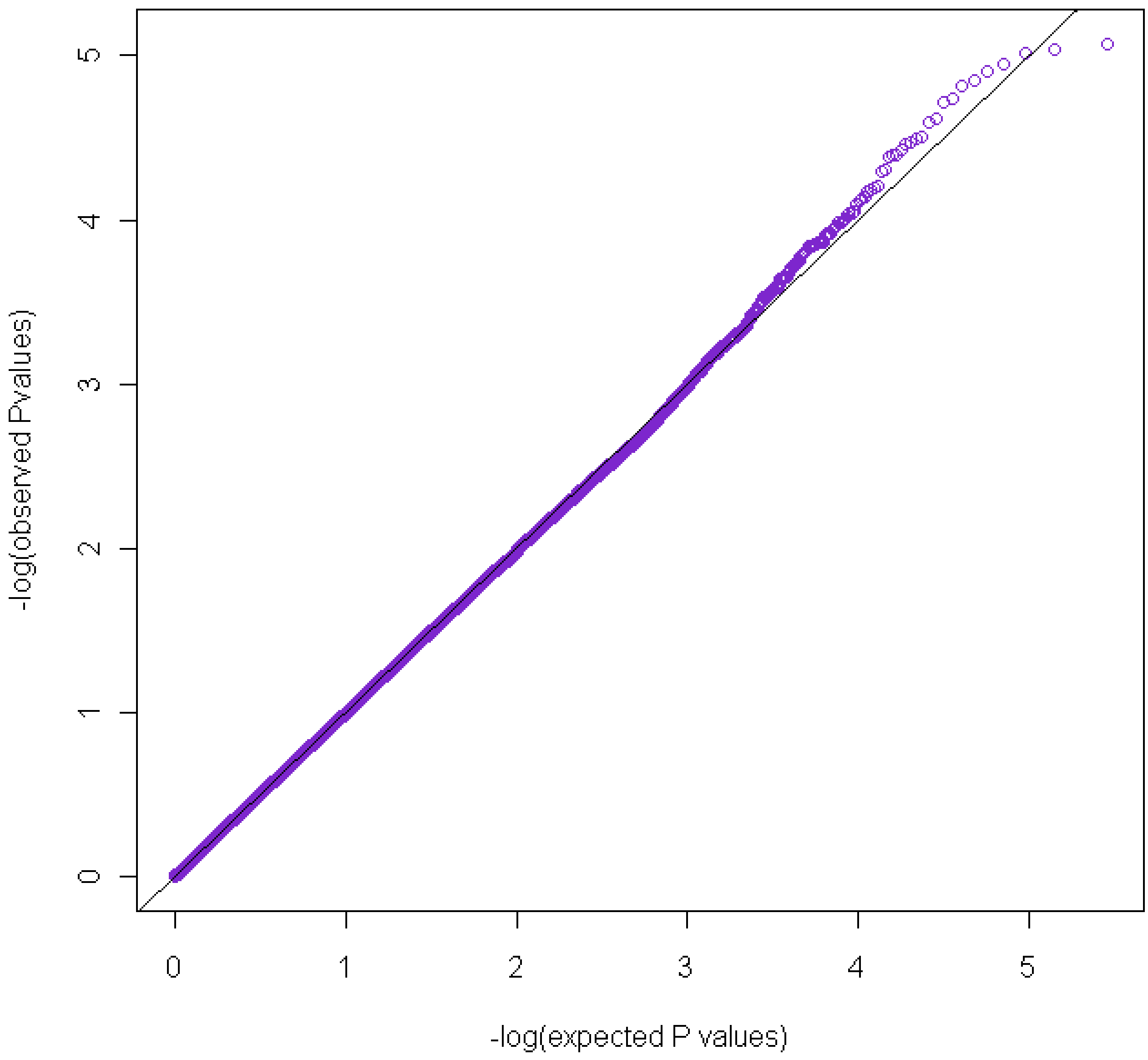

Supplementary Figure 3. Quantile-quantile plot for 285,984 SNP trend tests, adjusted for population stratification using five principal components (Finnish subjects only). Genomic control inflation factor (λ) = 1.0137.

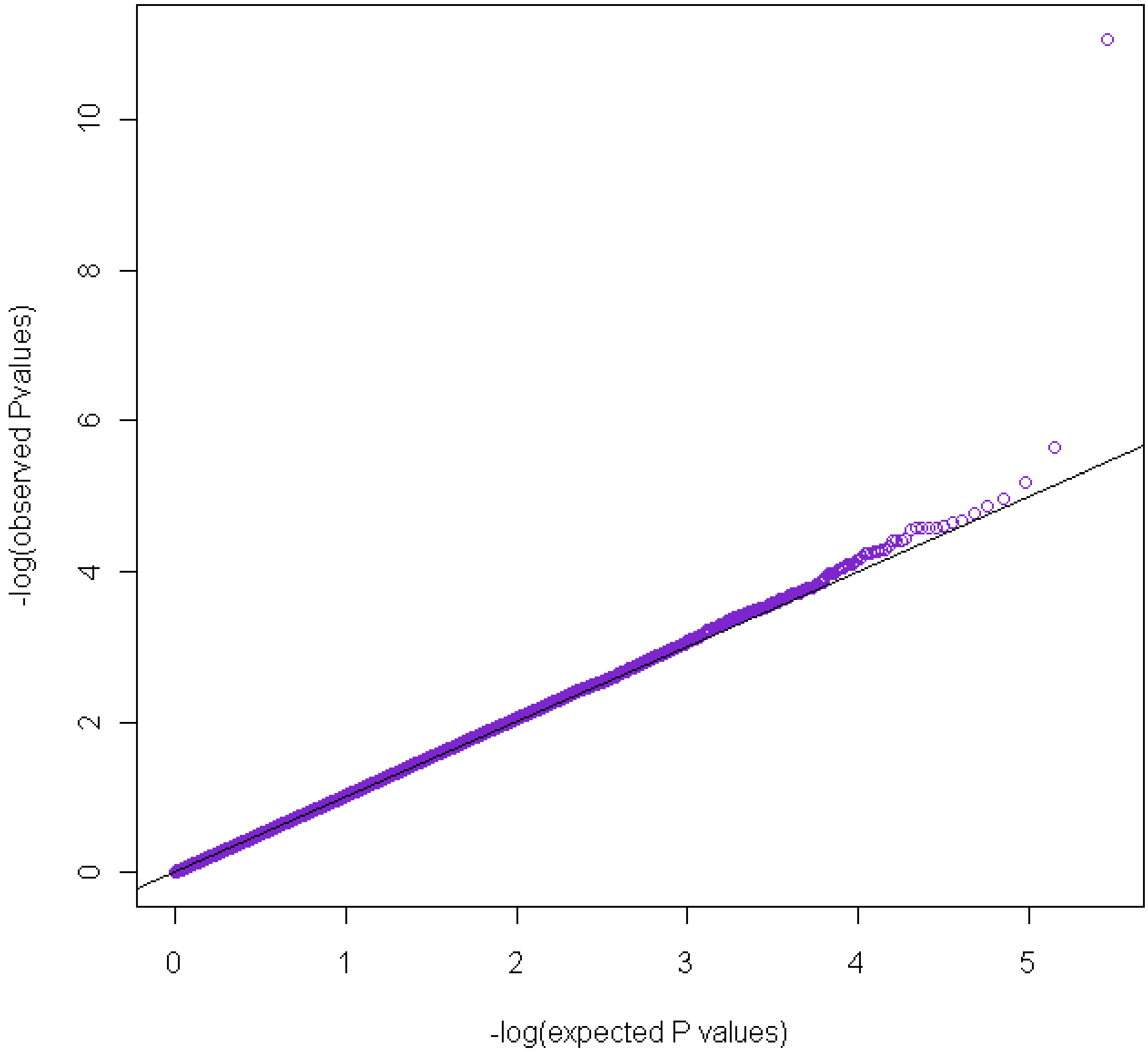

Supplementary Figure 4. Quantile-quantile plot for 285,984 SNP trend tests, adjusted for population stratification (combined analysis of Swedish and Finnish subjects). Genomic control inflation factor (λ) = 1.0218.

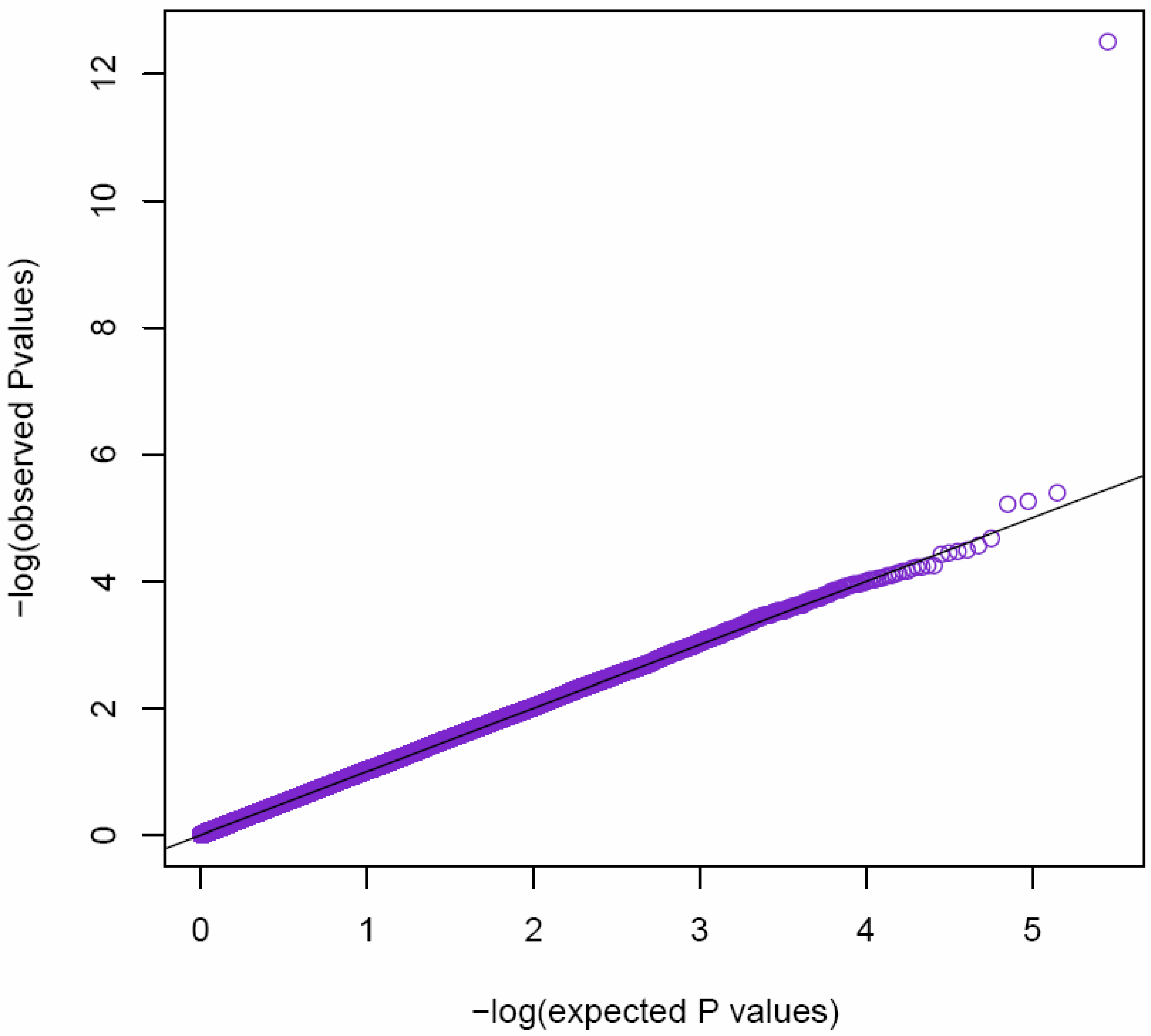

Supplement: Additional file 2 — Supplementary figures. Supplementary Figure 1. Scree plot of log-transformed Eigen values. Vertical dashed lines indicate three and five PCs taken to correct for population stratification within the Swedish and Finnish populations respectively. Supplementary Figure 2. Quantile-quantile plot for 285,984 SNP trend tests, adjusted for population stratification using three principal components (Swedish subjects only). Genomic control inflation factor (λ) = 1.0140. Supplementary Figure 3. Quantile-quantile plot for 285,984 SNP trend tests, adjusted for population stratification using five principal components (Finnish subjects only). Genomic control inflation factor (λ) = 1.0137. Supplementary Figure 4. Quantile-quantile plot for 285,984 SNP trend tests, adjusted for population stratification (combined analysis of Swedish and Finnish subjects). Genomic control inflation factor (λ) = 1.0218. [file bcr2772-S2.pdf]
