# Supplementary material for: A genome-wide association scan on estrogen receptor-negative breast cancer
Source: Breast Cancer Res. 2010 Nov 9;12(6):R93. doi: 10.1186/bcr2772 (PMC3046434; doi:10.1186/bcr2772)
Supplement: Additional file 3 — Supplementary tables. Table S1. Association analysis results of reclustered SNPs. Table S2. Association results of top hits in the combined analysis, with corresponding MAF, ORs and P values within the Swedish and Finnish populations. * denotes the five SNPs selected for validation in SEARCH and RBCS. Table S3. Power to detect single marker effects in genome-wide association study. [file bcr2772-S3.pdf]

Table S1. Association analysis results of reclustered SNPs.

CHR	SNP	BP	A1	NMISS	OR	SE	L95	U95	P
5	rs4549482	54015758	G	1705	1.052	0.2219	0.6812	1.626	0.8179
1	rs1984492	1,56E+08	G	1688	0.8319	0.185	0.5788	1.196	0.3198
8	rs1389545	1,2E+08	A	1703	0.8412	0.1718	0.6007	1.178	0.3142
8	rs3748648	62014020	G	1718	0.6427	0.1568	0.4727	0.8738	0.004798

CHR	Chromosome
SNP	Single nucleotide polymorphism (SNP) identifier
BP	Physical position (base-pair)
A1	Tested allele (minor allele by default)
NMISS	Number of non-missing individuals included in analysis
OR	Odds ratio
L95	Lower bound of 95% confidence interval for odds ratio
U95	Upper bound of 95% confidence interval for odds ratio
P	P-value

Table S2. Association results of top hits in the combined analysis, with corresponding MAF, ORs and P values within the Swedish and Finnish populations. * denotes the five SNPs selected for validation in SEARCH and RBCS. SNPs rs7039994 and rs12000794, located 106310 base pairs away from each other on chromosome 9, were found to be in high LD (r2=0.797; D'=0.952). The former was kept and validated in the SEARCH dataset as its associated *P* value was smaller and it was in closer proximity to coding regions (downstream of *INVS|TEX10*). SNP rs3777218 was selected over rs11882068 due to a better regional signal peak. Of the five SNPs forwarded for validation, rs4726078 could not be designed and was replaced by rs10952315 (r2=0.977 in Centre d'Etude du Polymorphisme Humain (CEPH) from Utah (CEU) HapMap samples).

					Swedish study population				Finnish study population				Combined				Validation		
					153 cases, 1414 controls				464 cases, 3169 controls				617 cases, 4583 controls				1011 cases, 7604 controls		
SNP	CHR		GENE	A1	MAF Case/Control		OR (L95-U95)	P	MAF Case/Control		OR (L95-U95)	P	MAF Cases/Controls		OR (L95-U95)	P	MAF	OR (L95-U95)	P
rs361147	4	*	n/a	C	0.33	0.41	0.69 (0.54-0.89)	4.66E-03	0.37	0.51	0.57 (0.49-0.67)	8.46E-12	0.36	0.48	0.6 (0.53-0.69)	3.13E-13	0.41	0.97 (0.88-1.07)	0.5767
rs7039994	9	*	INVS|TEX10	A	0.24	0.16	1.61 (1.21-2.13)	9.98E-04	0.16	0.13	1.44 (1.16-1.79)	9.56E-04	0.18	0.14	1.5 (1.26-1.78)	3.95E-06	0.15	1.00 (0.88-1.14)	0.9613
rs12000794	9		n/a	A	0.26	0.18	1.61 (1.22-2.12)	7.94E-04	0.17	0.15	1.41 (1.14-1.74)	1.48E-03	0.19	0.16	1.48 (1.25-1.75)	5.40E-06	--	-- (-- - --)	--
rs6993922	8	*	n/a	G	0.12	0.06	2.17 (1.47-3.22)	1.01E-04	0.08	0.05	1.53 (1.14-2.06)	4.69E-03	0.09	0.05	1.73 (1.36-2.20)	6.18E-06	0.08	1.02 (0.86-1.21)	0.8477
rs4726078	7	*	PRKAG2	G	0.31	0.4	0.67 (0.52-0.87)	2.06E-03	0.3	0.32	0.78 (0.66-0.92)	3.02E-03	0.3	0.35	0.74 (0.65-0.85)	2.79E-05	0.39	1.03 (0.93-1.13)	0.6115
rs11882068	19		NLRP9	G	0.17	0.13	1.33 (0.97-1.83)	7.62E-02	0.09	0.05	1.78 (1.34-2.37)	6.65E-05	0.11	0.08	1.56 (1.27-1.92)	3.25E-05	--	-- (-- - --)	--
rs3777218	5	*	RHOBTB3	A	0.52	0.44	1.38 (1.09-1.75)	8.07E-03	0.56	0.5	1.28 (1.10-1.49)	1.23E-03	0.55	0.48	1.32 (1.16-1.50)	3.32E-05	0.47	0.98 (0.89-1.07)	0.6131

SNP	Single nucleotide polymorphism (SNP) identifier
CHR	Chromosome
A1	Tested allele (minor allele by default)
MAF	Minor allele frequency
OR	Odds ratio
L95	Lower bound of 95% confidence interval for odds ratio
U95	Upper bound of 95% confidence interval for odds ratio
P	P-value

Table S3. Power to detect single marker effects in genome-wide association study.

	OR = 1.05		OR = 1.30	
	100 cases	500 cases	100 cases	500 cases
MAF = 0.05				
P < 0.01	0.0104	0.0122	0.0251	0.1110
P < 0.05	0.0513	0.0566	0.0930	0.2729
P < 0.10	0.1020	0.1098	0.1616	0.3871
P < 0.20	0.2026	0.2129	0.2791	0.5333
P < 0.30	0.3028	0.3138	0.3836	0.6332
P < 0.40	0.4027	0.4135	0.4807	0.7100
P < 0.50	0.5025	0.5122	0.6683	0.9367
MAF = 0.2				
P < 0.01	0.0115	0.0176	0.0684	0.4421
P < 0.05	0.0544	0.0724	0.1924	0.6809
P < 0.10	0.1065	0.1326	0.2916	0.7839
P < 0.20	0.2087	0.2425	0.4317	0.8747
P < 0.30	0.3093	0.3453	0.5370	0.9186
P < 0.40	0.4090	0.4439	0.6237	0.9445
P < 0.50	0.5082	0.5398	0.6990	0.9614
MAF = 0.5				
P < 0.01	0.0123	0.0221	0.1017	0.6326
P < 0.05	0.0568	0.0847	0.2563	0.8301
P < 0.10	0.1101	0.1500	0.3680	0.8979
P < 0.20	0.2133	0.2647	0.5136	0.9488
P < 0.30	0.3142	0.3685	0.6149	0.9699
P < 0.40	0.4139	0.4662	0.6939	0.9810
P < 0.50	0.5126	0.5599	0.7593	0.9876
